# Groundwater oxygen isotope anomaly before the M6.6 Tottori earthquake in Southwest Japan

**DOI:** 10.1038/s41598-018-23303-8

**Published:** 2018-03-19

**Authors:** Satoki Onda, Yuji Sano, Naoto Takahata, Takanori Kagoshima, Toshihiro Miyajima, Tomo Shibata, Daniele L. Pinti, Tefang Lan, Nak Kyu Kim, Minoru Kusakabe, Yoshiro Nishio

**Affiliations:** 1 Atmosphere and Ocean Research Institute, The University of Tokyo, Kashiwa, Chiba, 277-8564 Japan; 2 Institute for Geothermal Sciences, Kyoto University, Beppu, Oita, 874-0903 Japan; 3 GEOTOP & Département des sciences de la Terre et de l’atmosphère, Université du Québec à Montréal, Montreal, H3C 3P8 Canada; 4 Department of Geosciences, National Taiwan University, Taipei, 10617 Taiwan; 5 Korea Polar Research Institute, 26 Songdomirae-ro, Yeonsu-gu, Incheon, 21990 Korea; 6 Graduate School of Integrated Arts and Sciences, Kochi University, Kochi, 783-8502 Japan

## Abstract

Geochemical monitoring of groundwater in seismically-active regions has been carried out since 1970s. Precursors were well documented, but often criticized for anecdotal or fragmentary signals, and for lacking a clear physico-chemical explanation for these anomalies. Here we report – as potential seismic precursor – oxygen isotopic ratio anomalies of +0.24‰ relative to the local background measured in groundwater, a few months before the Tottori earthquake (M 6.6) in Southwest Japan. Samples were deep groundwater located 5 km west of the epicenter, packed in bottles and distributed as drinking water between September 2015 and July 2017, a time frame which covers the pre- and post-event. Small but substantial increase of 0.07‰ was observed soon after the earthquake. Laboratory crushing experiments of aquifer rock aimed to simulating rock deformation under strain and tensile stresses were carried out. Measured helium degassing from the rock and ^18^O-shift suggest that the co-seismic oxygen anomalies are directly related to volumetric strain changes. The findings provide a plausible physico-chemical basis to explain geochemical anomalies in water and may be useful in future earthquake prediction research.

## Introduction

Hydro-geochemical precursors of major earthquakes have attracted the attention of researchers worldwide, because they are not entirely unexpected^1,2^. Monitoring groundwater has been carried out for earthquake prediction in USA, Japan, China and Italy since 1970s^3–5^. Anomalies of noble gases such as radon and helium have been documented^6^. However, predicting anomalies spatially and temporally is difficult because of the nonlinear nature of earthquake dynamics^7^, and lack of knowledge on the physico-chemical behavior of those elements during seismic activity. Critical review suggests that most of the observed precursor signals are anecdotal or fragmentary^8^ and they did not meet the criteria for a robust assessment^3^. Most authors have recognized that crustal deformation processes are responsible for the observed anomalies, while actual pre-seismic strains may be too small to be detected^9^.

Variation of oxygen isotopic ratios (^18^O/^16^O) of groundwater is generally attributable to evaporation of meteoric water^10^ together with ^18^O-shift by water-rock interaction^11^. A few reports indicated changes of oxygen isotopes before earthquakes^12–14^. However, all data were not clearly related to strain changes induced by crustal deformation, because their observation wells were too far from epicenters. So, unusual and local enhancement of sensitivity for tiny strain change was required to explain these anomalies, which often led to the conclusion that geochemical precursors are elusive^7,9^. Here we report a groundwater geochemical anomaly detected prior to the 2016 Tottori earthquake (M6.6) in southwest Japan. We observed oxygen isotopic changes in groundwater at an observation well, located 5 km away from the epicenter, much closer than in the other studies^3,6,12–14^. We try to understand the physical processes behind this anomaly, by carrying out preliminary laboratory experiments with water-rock interaction by a frozen crush method.

## Results

### The 2016 Tottori earthquake

**Figure 1:**
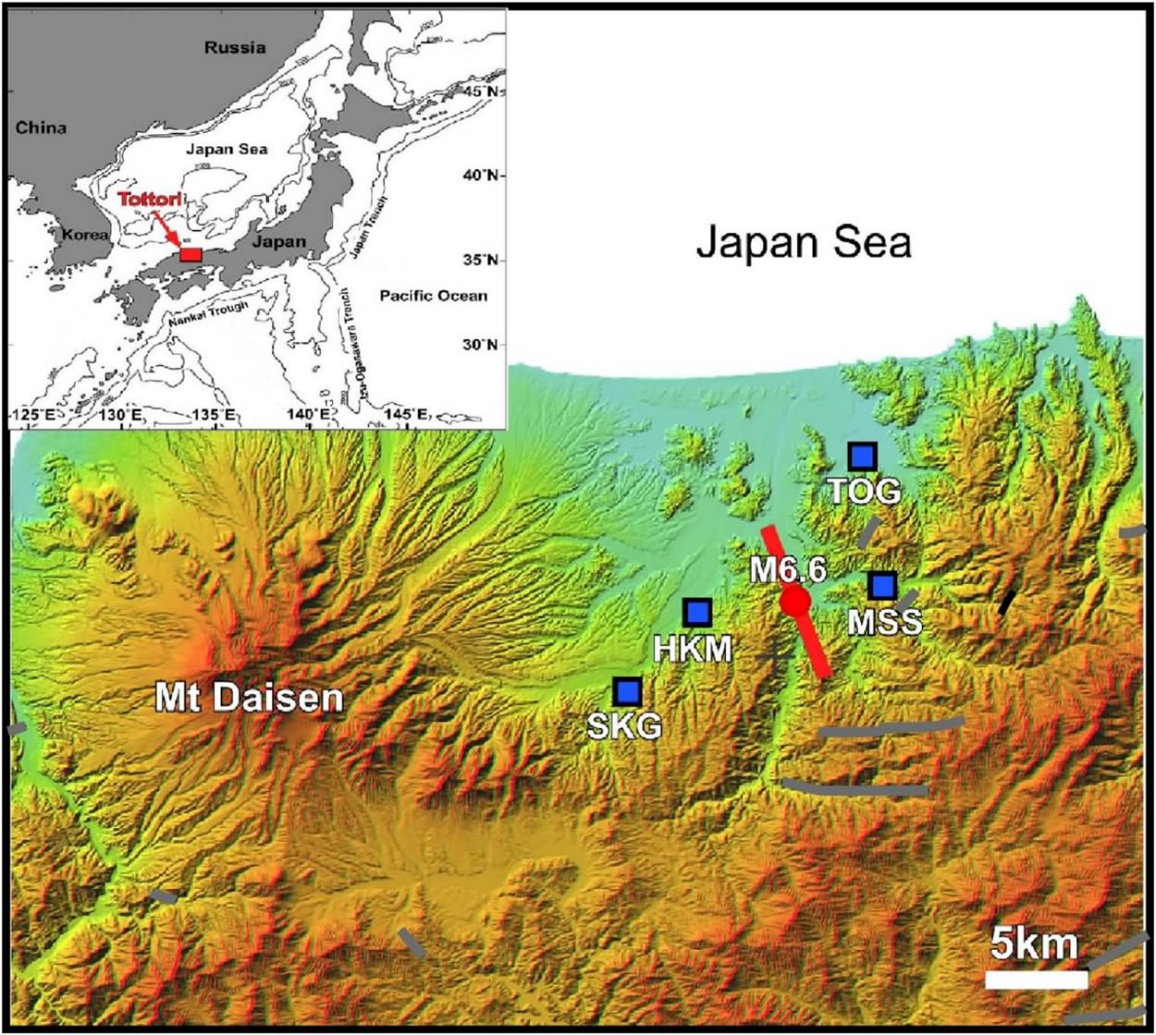
Sampling sites of hot spring and groundwater samples in central Tottori region (Southwest Japan) together with epicenter of the main shock (M6.6) of the 2016 Tottori earthquake. Red thick line represents the active fault emplacement as defined by aftershocks^17^. Gray lines show the other active faults in the region originally created by Tomo Shibata. TOG, MSS, and SKG denote Togo, Misasa, Sekigane hot spring, respectively, where we have collected helium samples. HKM shows Hakusan Meisui groundwater well, where mineral water samples were bottled. The base map was generated by the Digital Japan Portal Web Site, Geospatial Information Authority of Japan (http://maps.gsi.go.jp/#10/35.366096/133.778458/&base_grayscale=1&ls=std%7Canaglyphmap_color&blend=0&disp=01&lcd=anaglyphmap_color&vs=c0j0l0u0t0z0r0f0&d=v) and modified by Yuji Sano. Inset map was created by Yuji Sano.

Earthquakes in subduction zones such as Japan can be classified into two categories: inshore shallow crustal earthquakes with magnitude 6–7^15^ and offshore megathrusts with magnitude up to 9 as observed in descending plate boundaries^16^. Even though the inland earthquakes are smaller in magnitude and have longer recurrence intervals, they cause serious damage to communities. The 2016 Tottori earthquake (M6.6, Japan Meteorological Agency scale) occurred in central Tottori prefecture on Honshu Island, Southwest Japan (Fig. 1) at 14:07 (JST) on 21 October 2016^17^. Severe ground shaking injured 32 people and damaged over 15,000 houses^18^. It was an inland crustal earthquake at a depth of approximately 10 km caused by a strike-slip fault with a compression axis in a WNW-ESE direction. After the M6.6 event, aftershocks were continuously observed in a region extending about 10 km in NNW-SSE direction (Fig. 1)^17^.

### Sampling site of groundwater

To document the geochemical background of groundwater in central Tottori before and after the earthquake, we collected commercial bottled water covering a period of 22 months in between the Tottori earthquake event. This approach was suggested by a study of groundwater chemical variations prior and after the M7.2 Kobe earthquake in 1995^19^. Cretaceous-Paleogene granitoids are exposed in the Tottori region^20^ and high-quality mineral water is available from the altered granite layers. All samples were collected at the Hakusan Meisui (HKM) site, located approximately 5 km west of the Tottori earthquake epicenter (Fig. 1). There is a drilled well up to 1500m-deep. Groundwater pumped from a 240m-deep aquifer with approximately 15 °C is filtered and sealed in polyethylene terephthalate bottles and distributed on the market. Twenty-seven samples were bottled water distributed from September 2015 to July 2017. Hydrogen (D/H) and oxygen (^18^O/^16^O) isotopes of water were measured by a cavity ring-down spectroscopy (L2120-i Analyzer, PICARRO Co. Ltd) without any preprocessing. Observed hydrogen and oxygen isotopic ratios were calibrated against our in-house water standard and converted into the conventional V-SMOW unit, expressed as per mil (‰) in STable 1. Instrumental errors of δ^18^O and δD values were less than 0.05‰ and 0.3‰ at 2σ.

### Secular variations of oxygen isotopes

**Figure 2:**
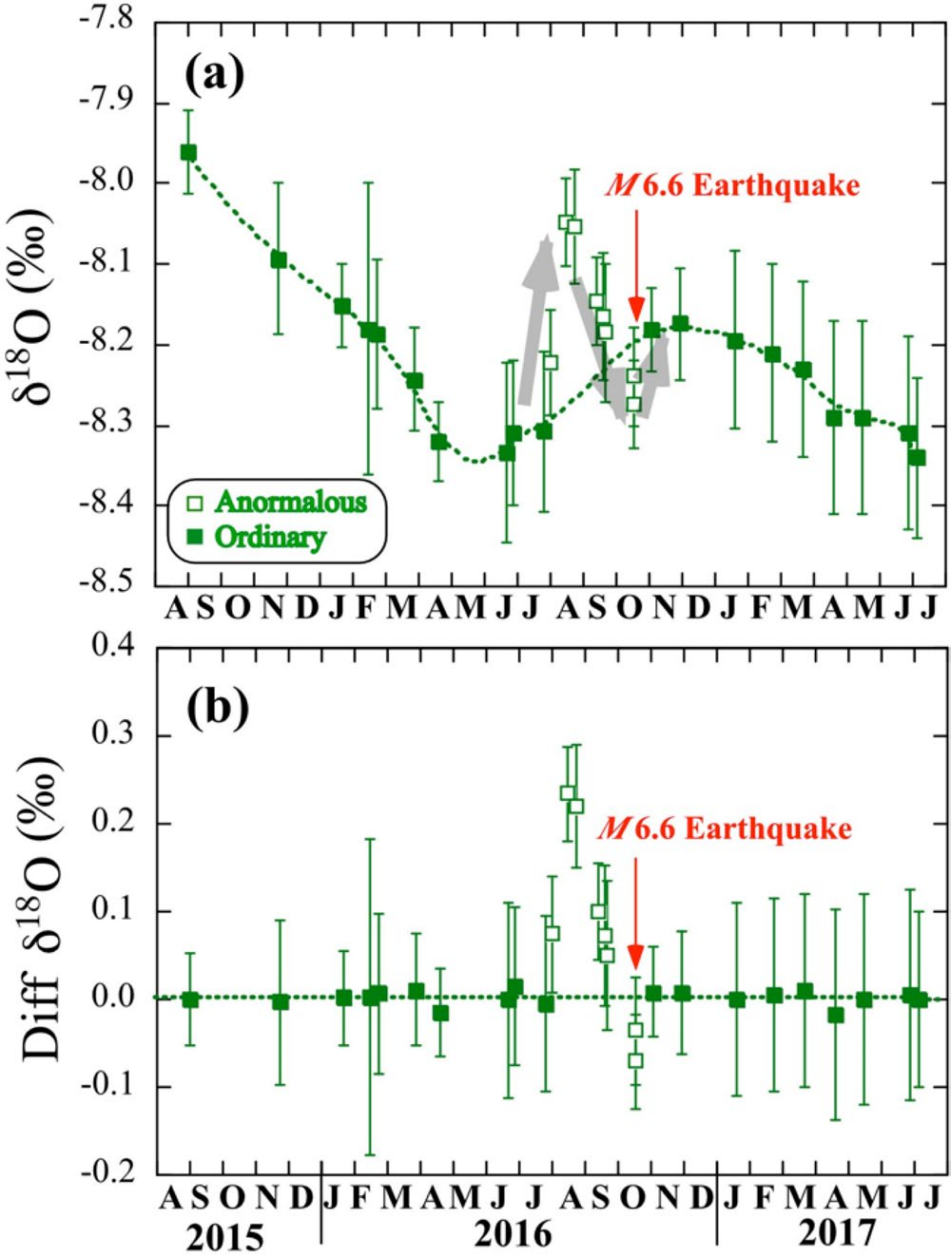
Temporal variation of δ^18^O values of deep groundwater at the HKM site from September 2015 to July 2017. (**a**) Background regional variation (dotted curve) is estimated using a spline function method, by masking anomalous data (open squares) from August 2^nd^ to October 18^th^ 2016. Error assigned to the symbol is two sigma. Red arrow indicates the M6.6 earthquake event. (**b**) Temporal variation of difference between observed and calculated δ^18^O values. Error assigned to the symbol is 2σ. Red arrow shows the M6.6 earthquake event.

Figure 2a shows the secular variation of ^18^O/^16^O ratios of groundwater from September 2015 to July 2017. The δ^18^O values with a typical error of 0.05–0.12‰ (at 2σ in STable 1) decrease gradually from −7.96‰ on September 1^st^ 2015 to −8.33‰ on June 21^st^ 2016. They then increase to −8.17‰ on November 29^th^ 2016 – except for irregular variation of the data a few months before the M6.6 event (open squares in Fig. 2a) – and then they decrease slowly to −8.34‰ on July 6^th^ 2017. It is important and critical for us to note that two samples collected on August 16^th^ and 23^rd^ 2016 show significantly heavier δ^18^O values of −8.05‰ than those of about −8.20‰ before and after the middle August. These oxygen variations, lasting three months until October 21^st^ 2016, might represent a geochemical anomaly related to the M6.6 Tottori earthquake.

## Discussion

Most of the geochemical precursors in water reported in literature^1,8^ have been rejected as fragmentary. Among a few convincing pieces of evidence, there is the groundwater radon anomaly^21^ and the chlorine and sulfate variations in mineral water^19^ prior to the 1995 Kobe earthquake in Japan. Chlorine contents started increasing about 4 months before the Kobe earthquake, while radon increase began 3 months before. Inspired by the anomalous period of 3–4 months in Kobe earthquake, we masked our δ^18^O data from August 2^nd^ to October 18^th^ 2016 and calculated a general trend for the temporal variation of δ^18^O values using a spline function method. Estimated variation is shown by the dotted curve in Fig. 2a. The peak and bottom are observed in November and May 2016. There is no clear explanation of general trend; a monotonic decrease of δ^18^O values from August 2015 to April 2016 and November 2016 to July 2017, perhaps due to local phenomena. This should be clarified in future work with much longer observation.

We calculated the difference between the observed and the smoothed δ^18^O values. Figure 2b shows temporal variation of the δ^18^O differences. There are two anomalies of groundwater oxygen before the M6.6 event beyond the accuracy. One is an obvious positive peak with the maximum of +0.24 ± 0.05‰ (2σ) found on August 16^th^. The other is a small but substantial negative peak with the minimum of −0.07 ± 0.05‰ (2σ) on October 18^th^, three days before the M6.6 event. The latter indicates that the co-seismic increase of the δ^18^O value is 0.07‰.

**Figure 3:**
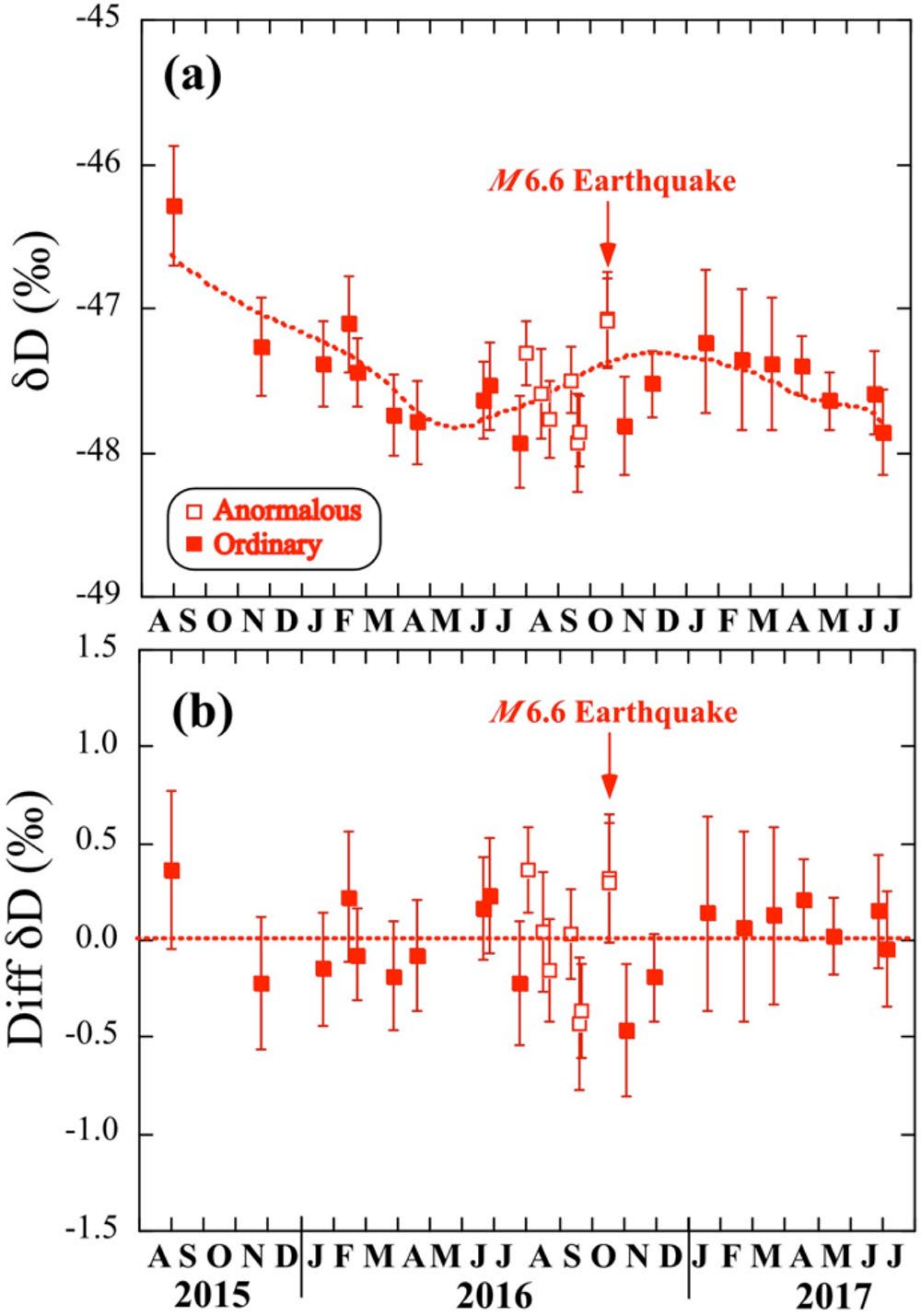
Emporal variation of δD values of deep groundwater at the HKM site from September 2015 to July 2017. (**a**) Background regional variation (dotted curve) is estimated by that of δ^18^O value (in Fig. 2a) and linear correlation between δ^18^O and δD values (in SFig. 1). Error assigned to the symbol is 2σ. (**b**) Temporal variation of difference between observed and calculated δD values. Error assigned to the symbol is 2σ.

To clarify the mechanism controlling the δ^18^O variations, the δD values were also measured and reported on a Craig’s plot^10^ together with the local meteoric water line (LMWL) with a slope of 8 and a d-excess of 18 (ref^22^). The δ^18^O and δD values of the water samples are different from local precipitation at Misasa and depart from the LMWL with a slope of ca. 3.1 calculated by a least-square method (SFig. 1). To explain this departure, evaporation^10^ or δ^18^O-shift by water-rock interaction^11^ can be considered. Observed δD values of groundwater decrease gradually from −46.3‰ in September 2015 to −47.8‰ in April 2016 and then increase to −47.2‰ in January 2017 with some irregular variations (Fig. 3a). There is no significant change of δD values before the 2016 Tottori earthquake. Based on the relationship between δ^18^O and δD values of groundwater and the estimated trend of the δ^18^O temporal variation, a general trend for the δD variation was also traced (dotted curve in Fig. 3a). Then we can assess the secular variation of the difference between observed and calculated δD values. Note that there is no anomalous change of the δD differences before the M6.6 event (Fig. 3b). On the other hand, there were small increases of chloride (Cl^−^) and sulfate (SO_4_^2−^) contents three months before the 2016 Tottori earthquake (SFig. 3 and STable 2), even though experimental errors are large. The anomalous trends are similar to those observed before the 1995 Kobe earthquake^21^, suggesting a common basis. Note that there is not significant temperature change of groundwater after the Tottori earthquake as mentioned by the mineral water company.

The most probable mechanism to cause δ^18^O enrichment without δD change of the groundwater is a ^18^O-shift caused by water-rock interaction^11^. Aquifer rocks in Tottori area are altered Cretaceous-Paleogene granitoids, whose δ^18^O values are ranging from +6.9‰ to +10.6‰ with the average of +9.0‰^23^. We have measured δ^18^O values of aquifer rocks, ranging from +8.20‰ to +8.56‰. Since observed groundwater shows δ^18^O values of about −8.2‰, much lower than those of aquifer rocks, a progressive isotopic equilibration of oxygen would make the groundwater δ^18^O values increase. On the other hand, the δD values of groundwater remain unchanged because the hydrogen content of granitic rocks is significantly low compared to that of water. Crustal deformation related to the 2016 Tottori earthquake may have enhanced water-rock interaction (i.e., by micro-fracturing and the resulting increase in rock surface area) in 240m-deep aquifer, induced ^18^O enrichment of water that was in contact with the fractured rocks and appeared as the precursor and co-seismic oxygen isotope anomalies (Fig. 2) together with slight increases of Cl^−^ and SO_4_^2−^ contents (SFig. 3).

Independent from stable isotope analysis of bottled mineral water as stated above, we have conducted a survey of noble gas in hot spring in the Tottori region. Groundwater helium isotopic variations were reported during the 2016 Kumamoto earthquake (M7.3) in Kyushu, southwest Japan^24^. The earthquake was of an inland crustal type produced along a strike-slip fault and with a shallow hypocenter (10 km), similar to the Tottori M6.6 event. Observed helium isotopes (^3^He/^4^He) variations, soon after the Kumamoto earthquake, were coupled with volumetric strain changes estimated using a fault model of crustal deformation. The relation between helium isotopes and volumetric strain can be explained by helium degassing that occurred during compressional loading of rocks as demonstrated in laboratory^25^. In this model, groundwater helium can be regarded as an effective strain gauge^24^. In order to verify this hypothesis for the Tottori event, we have collected hot spring samples at three sites, Sekigane (SKG), Misasa (MSS) and Togo (TOG) (Fig. 1) located close to the epicenter, soon after the event. Their ^3^He/^4^He and ^4^He/^20^Ne ratios were measured by a noble gas mass spectrometer^26^ (STable 3). This is entirely independent observation from δ^18^O analysis of groundwater at HKM (Fig. 2).

**Figure 4:**
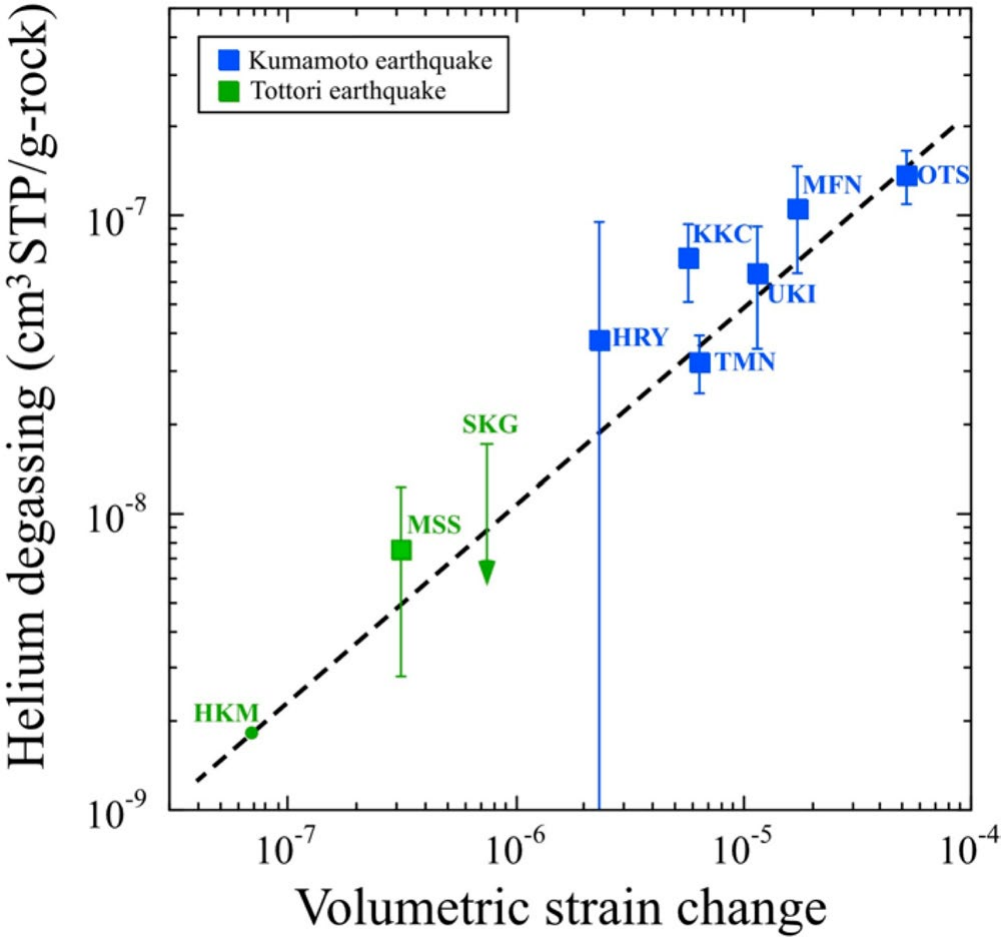
Correlation diagram between calculated volumetric strain change using a fault model of crustal deformation and helium degassing from a rock. Green and blue squares show Tottori and Kumamoto^23^ samples, respectively. Error assigned to the symbol is at 2σ. Dotted line is obtained by a least-squares fitting of the Kumamoto data^23^. MSS, STG, and HKM denote Misasa, Sekigane hot spring, and Hakusan-Meisui groundwater well, respectively in Tottori region. Arrow shows the upper limit. MFN, OTS, KKC, UKI, TMN, and HRY denote Mifune, Otsu, Kikuchi, Ueki, Tamana and Hirayama groundwater well in Kumamoto region. Details are given in STable 3.

Following the method that was developed to explain helium variations after the Kumamoto earthquake^24^, the amount of radiogenic helium degassed from Tottori area was estimated at two sites, Sekigane (SKG) and Misasa (MSS) (STable 3). Results were compared with data before the 2016 Tottori earthquake^27^. Figure 4 shows the obtained relation between co-seismic volumetric strain changes estimated by a fault model of rock deformation^28^ and helium degassing by aquifer rocks using this new set of data obtained for Tottori and the previous one from the Kumamoto study^24^. A dotted line is obtained by a least square method on the 2/3 power law for volumetric strain changes^25^. Observed Misasa data (MSS in Fig. 1) agree with the best fit line within experimental error, even though the estimated strain change is one or two order of magnitude smaller than that estimated by using the Kumamoto data. The Sekigane data (SKG in Fig. 1) are also consistent with the line under the upper limit. Therefore the Tottori data can be explained by the hypothesis that groundwater helium reflects strain change during the earthquake^24^.

In order to combine two individual observations: δ^18^O anomaly of groundwater and helium isotopic change in hot springs in region close to the epicenter of the 2016 Tottori earthquake, we have conducted a preliminary experiment where rock was crushed in frozen water^29^ (see Method). There is no reference that showing oxygen and helium isotope anomalies at the same time related to a single seismic activity. Therefore this is the first attempt to show a linkage between the ^18^O-shift and helium release during the crush experiment. Estimated helium degassing of 4.5 × 10^−9^ ccSTP/g is corresponding to δ^18^O-shift of 0.36‰ by water-rock interaction. Then it is possible to calculate a conversion factor from helium degassing to ^18^O-shift by rock fracturing, which is 8.0 ± 6.9 × 10^7^‰/ccSTP/g (2σ). Co-seismic volumetric strain change was estimated to be 6.88 × 10^−8^ at the HKM site using the fault model^28^. This value can be converted into helium degassing of 1.8 × 10^−9^ ccSTP/g based on the 2/3 power law between strain change and helium degassing (see HKM in Fig. 3). If the result of the rock crushing experiment is applicable to the present case, the amount of helium degassing, 1.8 × 10^−9^ ccSTP/g would be converted into the δ^18^O-shift of 0.14 ± 0.12‰ (2σ). The calculated shift would be consistent with observed co-seismic increase of δ^18^O value, 0.07 ± 0.05‰ (2σ) within the experimental error. We can therefore conclude that the co-seismic groundwater oxygen anomaly would be related to the volumetric strain change.

On the other hand, the pre-seismic increase of the δ^18^O value, up to 0.24‰ observed from July to August 2016 (Fig. 2b) is approximately 3.4 times larger than the co-seismic change of 0.07‰. Then volumetric strain change of 2.3 × 10^−7^ (3.4 times of 6.88 × 10^−8^) should have been observed at the HKM site two months before the M6.6 earthquake. Unfortunately, there is no record of volumetric strain change to verify the estimated pre-seismic strain change in the area close to the HKM (Fig. 1). At the 1995 Kobe earthquake, pre-seismic strain change of 3–6 × 10^−6^ was observed by a multicomponent borehole strain meter at the Rokko-Takao station, 5 km away from the groundwater observation well where geochemical anomaly was reported^19^. Crustal deformation at the estimated level of 2.3 × 10^−7^ may have occurred in the region 5 km away from the epicenter before the Tottori earthquake. However further discussion is difficult, because there is no geophysical observation as well as helium anomaly data from July to November 2016.

## Conclusion

We found an increase of +0.24‰ in oxygen isotopes of groundwater a few months before a M 6.6 earthquake in October 21^st^ 2016. The signal would be equivalent with volumetric strain change of 2.3 × 10^−7^ estimated by a relation between helium and oxygen changes by rock crushing experiment in a laboratory and helium isotopic variation observed in hot springs after the earthquake. This suggests that monthly monitoring of groundwater δ^18^O value together with helium abundance in deep well, up to 1000 m may detect a possible strain change prior to inland earthquakes in subduction zones.

## Methods

### Oxygen and hydrogen isotope analysis

Oxygen and hydrogen isotopes of mineral water (HKM) and recovered water after a vacuum crushing experiment were measured by Cavity Ring-Down Spectroscopy (CRDS) using a L2120-i (Picarro Inc.) connected to an automatic injection system and high-precision vaporizer. Three working standards (SHOKO Science Co.,Ltd., SI Science Reference Material), calibrated against international reference material (VSMOW), were analyzed with samples.

### Helium isotope analysis

Helium abundance and isotopic compositions of hot spring water (MSS, SKG and TOG) and head space gas after crushing in a stainless-steel ball mill were measured by a Helix SFT mass spectrometer after separation and purification usung a vacuum line. The ^3^He/^4^He ratio was calibrated against our inhouse standard and expressed as Ra notation where Ra is the atmopsheric ratio of 1.38 × 10^−6^.

### Anion analysis

Chlorine and sulfate ions of groundwater water at the HKM site were measured by ion chromatography (ICS 1500, DIONEX Thermo Fisher Scientific) after removal of suspended fine particles by 0.20 μm syringe-mounted filter. The samples were calibrated by five working standards (Wako Pure Chemical Industries Ltd, Standard Solition) analyzed in the sequence of sample measurement.

### Frozen crush experiment

Two granitic rock samples recovered from a drilled core of the HKM site (Fig. 1) at depths of −240 m and −1500 m were provided by the company that supplied the water samples. The rock sample derived from 240 m deep is primary important because it is potential aquifer rock. There is not enough amount of the 240 m rock to reproduce experiment. So we measured 1500 m deep rock because it is similar granitic rock in the same place. These rock samples were put in a stainless-steel vacuum crusher with 1 cm^3^ of the mineral water and a stainless-steel ball. Water was frozen by cooling the ball mill into liquid nitrogen and head space gases were evacuated. Then, the ball mill was shaken 500 times and helium in rock was extracted by mechanical fracturing^29^. At first gas extracted by the crushing was introduced into vacuum purification line and helium abundance and isotopic composition were measured by a noble gas mass spectrometer after separation^26^. Then reacted water was recovered and the δ^18^O and δD values were measured by a cavity ring-down IR spectrometer. In this experiment, hot and cold blanks mean blank test with crushing and no crushing, respectively. These are not related to temperature, both are cooled by liquid nitrogen.

Measured helium abundances are higher than hot blank, while isotopic ratios show only an upper limit because of a tail effect of high HD background at mass number 3 (STable 4). In order to obtain the ^3^He/^4^He ratio, we conducted a vacuum crushing experiment without water. The dry experiment indicated helium release with radiogenic ^3^He/^4^He ratios with 0.15-0.71Ra. Average helium abundance is 4.5 × 10^−9^ ccSTP/g for the 240 m and 1500 m depth samples by wet experiment with water (STable 3). On the other hand, reacted water and hot blank water had much higher δ^18^O and δD values than those of the initial water, probably due to an evaporation effect^10^. It is possible to calculate amounts of ^18^O-shift by correcting δ^18^O change due to evaporation equation using the hot blank water values (SFig. 3). For example, observed δ^18^O and δD values of 1500 m sample are −5.00‰ and −29.28‰, respectively, after the crushing (STable 4). If we assume an evaporation effect based on the cold and hot blank samples, an estimated δ^18^O value of 1500 m sample becomes −5.43‰ at δD = −29.28‰. Then it is easy to calculate the ^18^O shifts of +0.43 ± 0.44‰ (2σ) for 1500 m sample. The 240 m sample shows ^18^O shifts of +0.30 ± 0.43‰ (2σ) in a similar way. When one takes error for the weighted mean of the two results, ^18^O shift due to crushing experiment becomes +0.36 ± 0.31‰ (2σ), which is statistically not zero and larger than instrumental error of less than 0.05‰.

## Electronic supplementary material

Supplementary Information
