# Supplementary material for: Groundwater oxygen isotope anomaly before the M6.6 Tottori earthquake in Southwest Japan
Source: Sci Rep. 2018 Mar 19;8:4800. doi: 10.1038/s41598-018-23303-8 (PMC5859118; doi:10.1038/s41598-018-23303-8)
Supplement: Supplementary file 1 — Supplementary Information [file 41598_2018_23303_MOESM1_ESM.pdf]

Supplementary Information

Groundwater oxygen isotope anomaly before the M6.6 Tottori earthquake in Southwest Japan

Satoki Onda[1], Yuji Sano[1*], Naoto Takahata[1], Takanori Kagoshima[1], Toshihiro Miyajima[1], Tomo Shibata[2], Daniele L. Pinti[3], Tefang Lan[4], Nak Kyu Kim[5], Minoru Kusakabe[5] & Yoshiro Nishio[6]

[1]Atmosphere and Ocean Research Institute, The University of Tokyo, Kashiwa, Chiba 277-8564, Japan.

[2]Institute for Geothermal Sciences, Kyoto University, Beppu, Oita 874-0903, Japan.

[3]GEOTOP & Département des sciences de la Terre et de l'atmosphère, Université du Québec à Montréal, Montreal H3C 3P8, Canada.

[4]Department of Geosciences, National Taiwan University, Taipei, Taiwan.

[5]Korea Polar Research Institute, 26 Songdomirae-ro, Yeonsu-gu, Incheon 21990, Korea

[6]Graduate School of Integrated Arts and Sciences, Kochi University, Kochi 783-8502, Japan

*Correspondence and requests for materials should be addressed to Y.S. (e-mail: ysano@aori.u-tokyo.ac.jp)

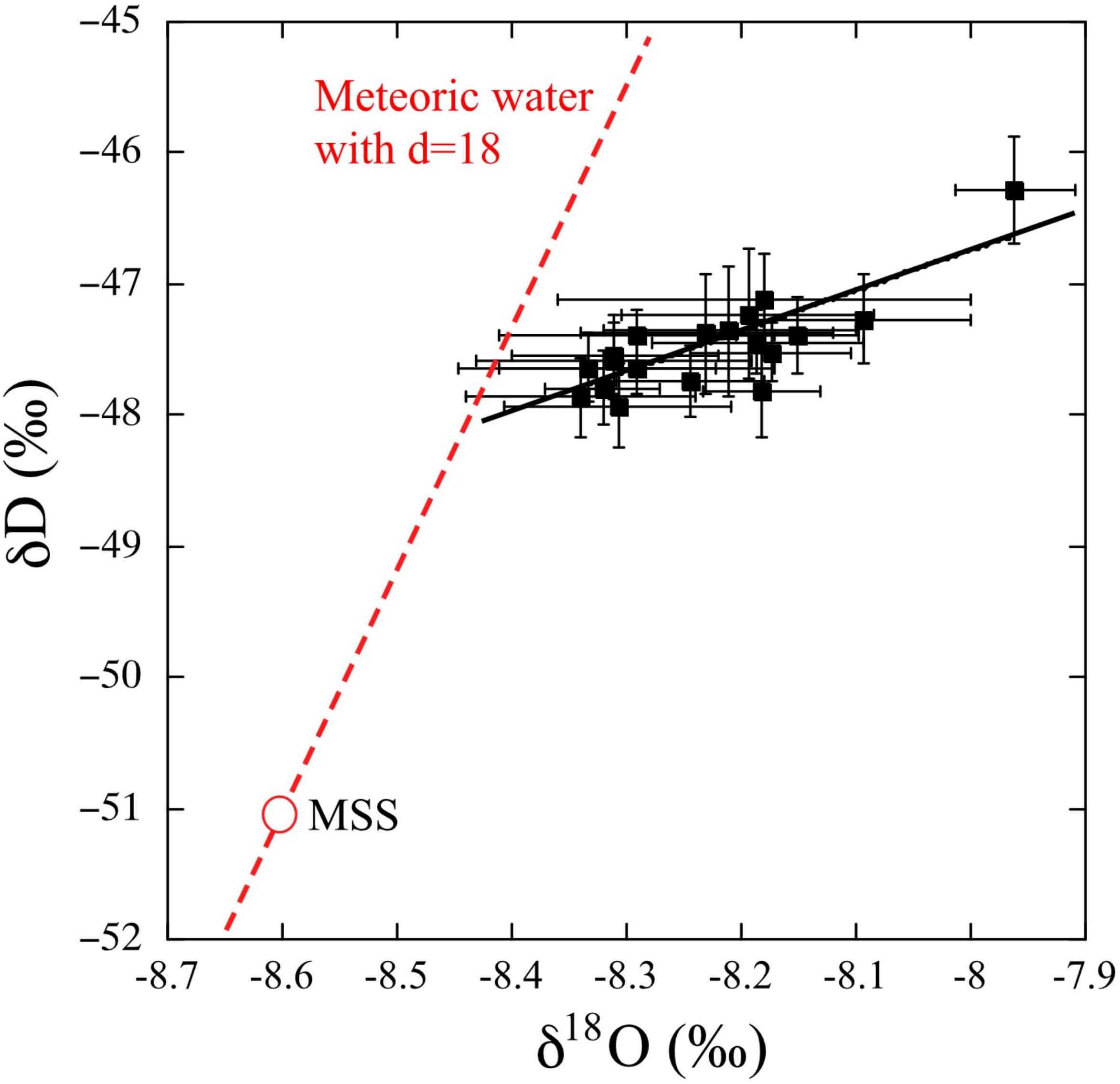

Supplementary Figure 1. A relationship between $\delta^{18}O$ and δD values of groundwater samples of ordinary period from Hakusan Meisui site. Fractionation of meteoritic water in the region (LMWL) is shown by a red dotted line with d=18 (ref. 22). MSS indicates precipitation data at Misasa. A solid line showes best fit by a least squre method with slope of 3.1. Error assigned to the simbol is 2σ.

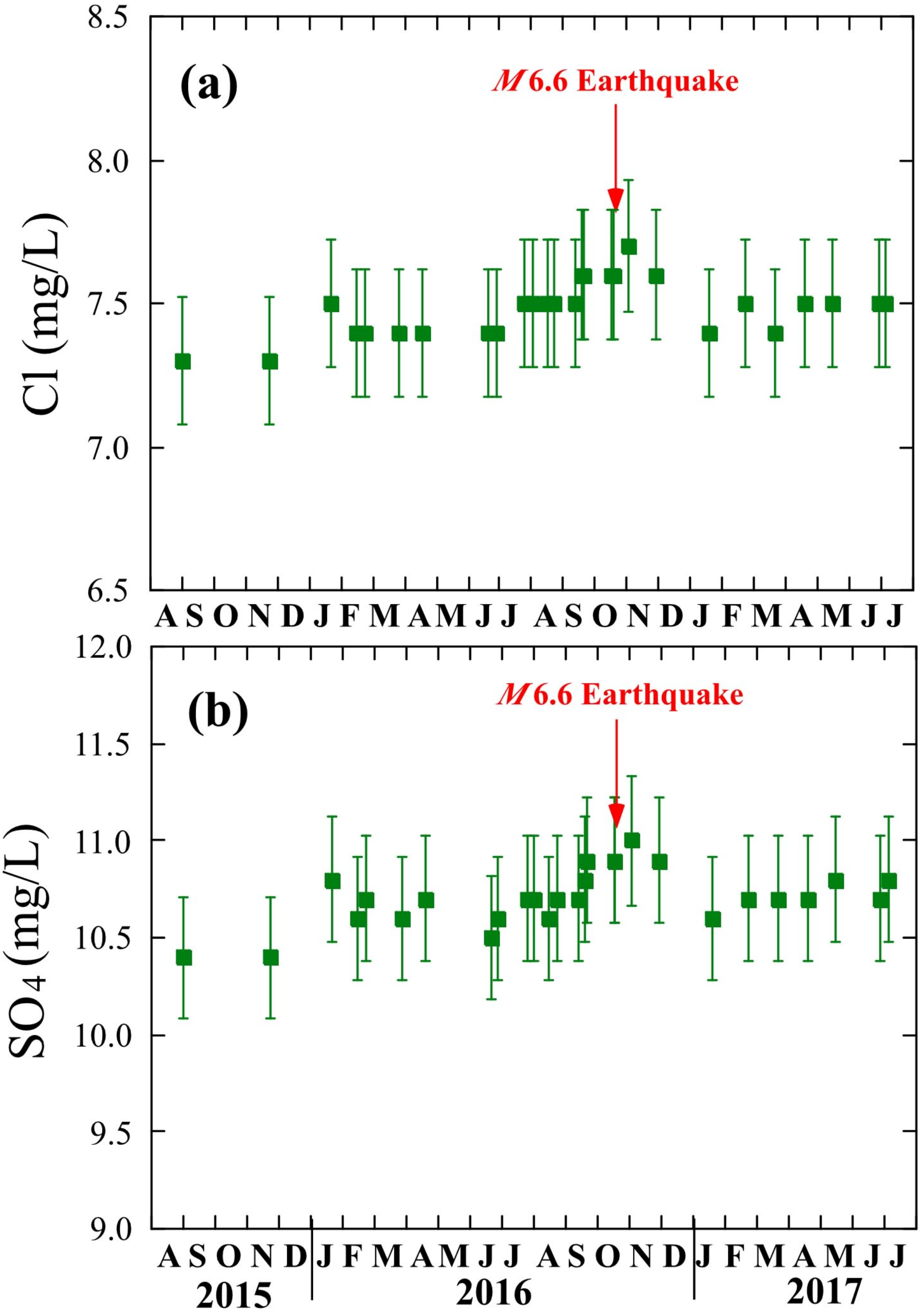

Supplementary Figure 2. Temporal variations of anions in deep groundwater at the Hakusan Meisui site from September 2015 to July 2017. (a) Those of chloride (Cl^-) and (b) sulfate (SO_4^{2-}). Error assigned to the symbol is 2σ. Both contents show small inreases three month before the M6.6 earthquake.

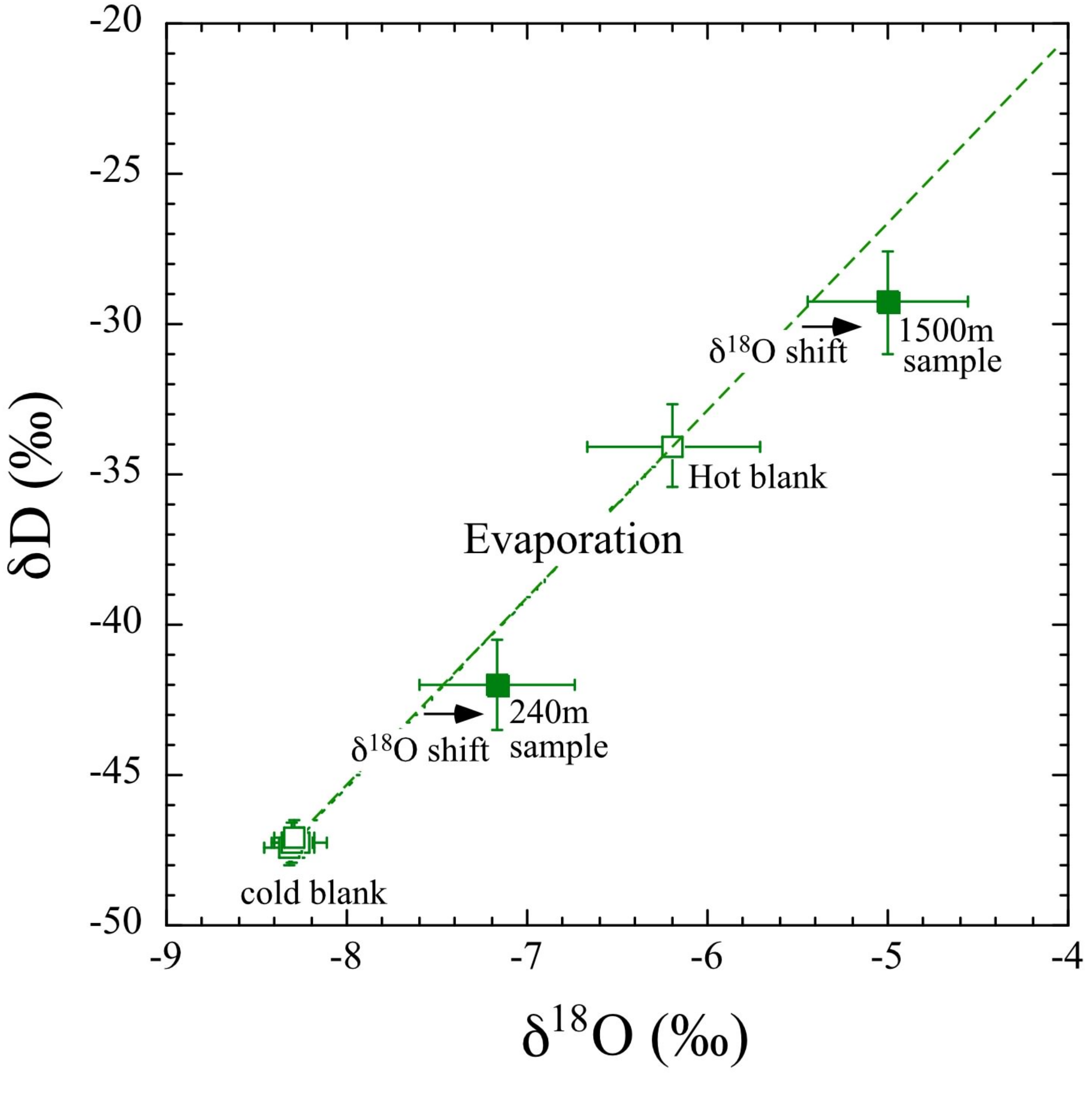

Supplementary Figure 3. A relationship between $\delta^{18}O$ and δD values of water samples recovered after frozen crushing experiments. Data of hot and cold blanks, 1500m and 240 m samples are shown with 2σ error. Evapolation line is calculated based on data of hot and cold blanks.

STable 1. Observed δ 18O and δ D values of groud water in central Tottori.

No	Date	δ 18O (‰)	err 2s	δ D (‰)	err 2s	est d18O (‰)	difference	est dD (‰)	difference
1	2015/9/1	-7.96	0.05	-46.29	0.41	-7.96	0.00	-46.61	0.33
2	2015/11/24	-8.09	0.09	-47.27	0.34	-8.09	0.00	-47.05	-0.22
3	2016/1/22	-8.15	0.05	-47.39	0.29	-8.15	0.00	-47.25	-0.13
4	2016/2/16	-8.18	0.18	-47.11	0.34	-8.18	0.00	-47.35	0.24
5	2016/2/23	-8.19	0.09	-47.44	0.24	-8.19	0.00	-47.38	-0.06
6	2016/3/28	-8.24	0.06	-47.74	0.28	-8.25	0.01	-47.59	-0.15
7	2016/4/19	-8.32	0.05	-47.79	0.29	-8.31	-0.01	-47.77	-0.02
8	2016/6/21	-8.33	0.11	-47.63	0.26	-8.33	0.00	-47.86	0.23
9	2016/6/28	-8.31	0.09	-47.54	0.30	-8.33	0.02	-47.83	0.29
10	2016/7/26	-8.31	0.10	-47.93	0.32	-8.30	0.00	-47.76	-0.17
11	2016/8/2	-8.22	0.07	-47.32	0.22	-8.30	0.07	-47.74	0.42
12	2016/8/16	-8.05	0.05	-47.59	0.31	-8.28	0.24	-47.69	0.10
13	2016/8/23	-8.05	0.07	-47.77	0.27	-8.27	0.22	-47.66	-0.11
14	2016/9/12	-8.15	0.06	-47.50	0.23	-8.25	0.10	-47.57	0.07
15	2016/9/19	-8.16	0.08	-47.94	0.34	-8.24	0.07	-47.54	-0.40
16	2016/9/21	-8.18	0.09	-47.85	0.24	-8.23	0.05	-47.53	-0.33
17	2016/10/17	-8.24	0.06	-47.08	0.33	-8.20	-0.04	-47.42	0.34
18	2016/10/18	-8.27	0.05	-47.09	0.31	-8.20	-0.07	-47.41	0.32
19	2016/11/2	-8.18	0.05	-47.82	0.34	-8.19	0.01	-47.37	-0.45
20	2016/11/29	-8.17	0.07	-47.52	0.23	-8.18	0.01	-47.34	-0.18
21	2017/1/19	-8.19	0.11	-47.23	0.50	-8.19	0.00	-47.39	0.16
22	2017/2/22	-8.21	0.11	-47.36	0.49	-8.21	0.00	-47.45	0.09
23	2017/3/22	-8.23	0.11	-47.38	0.46	-8.24	0.01	-47.51	0.13
24	2017/4/19	-8.29	0.12	-47.40	0.21	-8.27	-0.02	-47.61	0.21
25	2017/5/17	-8.29	0.12	-47.64	0.20	-8.29	0.00	-47.67	0.03
26	2017/6/29	-8.31	0.12	-47.59	0.29	-8.31	0.00	-47.74	0.15
27	2017/7/6	-8.34	0.10	-47.86	0.30	-8.34	0.00	-47.82	-0.04

STable 2. Observed concentrations of Cl and SO_4 in groudwater from central Tottori.

No	Date	Cl (mg/L)	SO_4 (mg/L)
1	2015/9/1	7.3	10.4
2	2015/11/24	7.3	10.4
3	2016/1/22	7.5	10.8
4	2016/2/16	7.4	10.6
5	2016/2/23	7.4	10.7
6	2016/3/28	7.4	10.6
7	2016/4/19	7.4	10.7
8	2016/6/21	7.4	10.5
9	2016/6/28	7.4	10.6
10	2016/7/26	7.5	10.7
11	2016/8/2	7.5	10.7
12	2016/8/16	7.5	10.6
13	2016/8/23	7.5	10.7
14	2016/9/12	7.5	10.7
15	2016/9/19	7.6	10.8
16	2016/9/21	7.6	10.9
17	2016/10/17	7.6	10.9
18	2016/10/18	7.6	10.9
19	2016/11/2	7.7	11.0
20	2016/11/29	7.6	10.9
21	2017/1/19	7.4	10.6
22	2017/2/22	7.5	10.7
23	2017/3/22	7.4	10.7
24	2017/4/19	7.5	10.7
25	2017/5/17	7.5	10.8
26	2017/6/29	7.5	10.7
27	2017/7/6	7.5	10.8

STable 3. Corrected $^3He/^4He$ ratios of before and after the 2016 earthquake, rock degassing helium and strain change.

Code	Sampling site	Corrected $^3He/^4He$ (Ra)		Helium degassing			Strain change
		Before Eq.*	After Eq.	(cm^3STP/g)			(DV/V)
Tottori							
MSS	Misasa	6.12	5.68	7.57E-09	±	4.74E-09	3.13E-07
SKG	Sekigane	4.78	4.84	<1.72E-08			7.37E-07
TOG	Togo	-	5.21				
Kumamoto							
MFN	Mifune	1.959	1.790	1.05E-07	±	4.12E-08	1.71E-05
OTS	Otsu	4.823	4.184	1.37E-07	±	2.76E-08	5.25E-05
KKC	Kikuchi	1.366	1.215	7.25E-08	±	2.09E-08	5.69E-06
UKI	Ueki	1.775	1.627	6.40E-08	±	2.81E-08	1.14E-05
TMN	Tamana	0.747	0.584	3.24E-08	±	7.10E-09	6.46E-06
HRY	Hirayama	0.645	0.626	3.84E-08	±	5.69E-08	2.30E-06

*: Data are refferred from Sano and Wakita[23] and Sano et al.[21].

STable 4. Vacuum crushing experiment of aquifer rock with ground water

	Sample rock (g)	δ18O (‰)	err 2s	δD (‰)	err 2s	O-18 shift (‰)	err 2s	4He (ccSTP/g)	3He/4He (Ra)
Original water-1		-8.26	0.11	-47.24	0.47				
Original water-2		-8.32	0.10	-47.45	0.51				
Cold blank-1		-8.30	0.10	-47.24	0.65				
Cold blank-2		-8.29	0.11	-47.04	0.56				
Hot blank-wet		-6.19	0.48	-34.04	1.36			1.1E-10	
Hot blank-dry								1.1E-10	
240m-wet	0.98	-7.16	0.43	-42.01	1.50	0.30	0.43	4.8E-09	<0.7
240m-dry	1.56							1.5E-08	0.15
1500m-wet	1	-5.00	0.44	-29.28	1.68	0.43	0.44	4.2E-09	<1.1
1500m-dry	1.5							2.1E-08	0.71
average-wet						0.36	0.31	4.5E-09	
